# Fading SARS-CoV-2 humoral VOC cross-reactivity and sustained cellular immunity in convalescent children and adolescents

**DOI:** 10.1186/s12879-023-08805-9

**Published:** 2023-11-22

**Authors:** Krystallenia Paniskaki, Sarah Goretzki, Moritz Anft, Margarethe J. Konik, Klara Lechtenberg, Melanie Vogl, Toni L. Meister, Stephanie Pfaender, Markus Zettler, Jasmin Jäger, Sebastian Dolff, Timm H. Westhoff, Hana Rohn, Ursula Felderhoff-Mueser, Ulrik Stervbo, Oliver Witzke, Christian Dohna-schwake, Nina Babel

**Affiliations:** 1 Department of Infectious Diseases, West German Centre of Infectious Diseases, University Hospital Essen, University Duisburg-Essen, Essen, Germany; 2 Center for Translational Medicine and Immune Diagnostics Laboratory, Medical Department I, Marien Hospital Herne, University Hospital of the Ruhr-University Bochum, Bochum, Germany; 3 Department of Pediatrics I, University Hospital Essen, University Duisburg-Essen, Essen, Germany; 4 Department of Pediatrics III, University Hospital Essen, University Duisburg-Essen, Essen, Germany; 5 Department of Molecular and Medical Virology, Ruhr-University Bochum, Bochum, Germany; 6 Medical Department I, Marien Hospital Herne, University Hospital of the Ruhr-University Bochum, Herne, Germany; 7 Berlin Institute of Health at Charité – University Clinic Berlin, BIH Center for Regenerative Therapies (BCRT) Berlin, Berlin, Germany

**Keywords:** SARS-CoV-2, T cells, Neutralizing antibodies, Adaptive immunity, Children

## Abstract

Cross-reactive cellular and humoral immunity can substantially contribute to antiviral defense against SARS-CoV-2 variants of concern (VOC). While the adult SARS-CoV-2 cellular and humoral immunity and its cross-recognition potential against VOC is broadly analyzed, similar data regarding the pediatric population are missing. In this study, we perform an analysis of the humoral and cellular SARS-CoV-2 response immune of 32 convalescent COVID-19 children (children), 27 convalescent vaccinated adults(C + V+) and 7 unvaccinated convalescent adults (C + V-). Similarly to adults, a significant reduction of cross-reactive neutralizing capacity against delta and omicron VOC was observed 6 months after SARS-CoV-2 infection. While SAR-CoV-2 neutralizing capacity was comparable among children and C + V- against all VOC, children demonstrated as expected an inferior humoral response when compared to C + V+. Nevertheless, children generated SARS-CoV-2 reactive T cells with broad cross-recognition potential. When compared to V + C+, children presented even comparable frequencies of WT-reactive CD4 + and CD8 + T cells with high avidity and functionality. Taking into consideration the limitations of study - unknown disease onset for 53% of the asymptomatic pediatric subjects, serological detection of SARS-CoV-2 infection-, our results suggest that following SARS-CoV-2 infection children generate a humoral SARS-CoV-2 response with neutralizing potential comparable to unvaccinated COVID-19 convalescent adults as well a sustained SARS-CoV-2 cellular response cross-reactive to VOC.

**Supplementary Information:**

The online version contains supplementary material available at 10.1186/s12879-023-08805-9.

## Key statement

Following SARS-CoV-2 infection children generate a humoral SARS-CoV-2 response with neutralizing potential comparable to unvaccinated COVID-19 convalescent adults as well a sustained robust SARS-CoV-2 cellular response cross-reactive to VOC, comparable to vaccinated convalescent adults with respect to magnitude and functionality.

**Supplementary Information:**

The online version contains supplementary material available at 10.1186/s12879-023-08805-9.

## Introduction

In contrast to other respiratory viruses [1, 2] children develop less severe SARS-CoV-2 infection [3, 4] despite bearing similar viral load compared to adults [5, 6]. However, relative to their risk of contracting disease, children and adolescents have been disproportionately affected by lockdown measures, taking in consideration school closures and limitation of their social and education activities [7–9].

Emerging data offer insight on the components of the pediatric immune system leading to reduced COVID-19 disease burden among children and young adults. Fialkowski et al. and Pierce et al. suggest that the absence in children of maladaptive immune responses that triggers the acute respiratory distress syndrome in older age groups [10, 11] are of advantage in case of COVID-19. On the contrary, independent studies underline the crucial role of innate immunity, which leads to viral elimination, without the need of a robust adaptive immune response [5, 12–16]. Several studies have shown that children are able to develop a robust neutralizing antibody response approximately a week after symptom onset [17–19] which is positively correlated with specific T and antigen-specific B cells and faster virus clearance [20–22].

Despite the approval of SARS-CoV-2 vaccines for children older than 6 months of age [23] and the emerging VOC, SARS-CoV-2 specific immune response in children against different VOC is understudied [24–27]. In this study, we perform an analysis of SARS-CoV-2 specific immune response of 32 convalescent COVID-19 children and 34 convalescent COVID-19 adults.

## Materials and methods

### Study participants

32 convalescent COVID-19 children (further referred as children) and 34 convalescent COVID-19 adults were recruited. A direct molecular sequencing of the viral variant responsible for the SARS-CoV-2 infection of each participant was not feasible, since we studied already convalescent COVID-19 subjects later in follow up. As the recruitment of the study participants took place between June 2020 and August 2021, we relied on temporal viral spreading taking into consideration the epidemiological trends in Germany between June 2020 and August 2021. According to epidemiological data, the majority of the study participants were infected with either the WT, alpha and/or delta variant. The omicron variant and its subvariants drove the pandemic in Germany since the end of November 2021, way past the end of the recruitment timeline [28]. Demographic and clinical characteristics are provided in Table 1.

**Table 1:** Demographic and clinical characteristics of the study cohorts

	children (N = 32)	C + V+ (N = 27)	C + V- (N = 7)	
Age years -median (range)* ^**1**^	11 (1–18)	47 (19–68)	46 (20–65)	
**Female gender N (%)**	12 (37.5)	17 (63)	5 (71)	
Time since COVID-19 Diagnosis (months)* ^**2**^ **median (range)**	6 (1–15)	12 (7–16)	9 (5–12)	
**COVID-19 Severity N (%)**				
*Asyptomatic-Mild*	32 (100)	25 (93)	7 (100)	
*Severe*	0	1 (3.5)	0	
*Critical*	0	1 (3.5)	0	
**Time since last antigenic contact** (months)* ^**3**^ **median (range)**	6 (1–15)	5 (4–8)	9 (5–12)	
**Number of mRNA vaccinations**				
1 N (%)	0	19 (70)	0	
2 N (%)	0	8 (30)	0	
Number of antigenic contacts	0	2 (2–3)	1	

*^1^ Children vs. C + V + p < 0.0001 two tailed unpaired t test

*^2^ Months from SARS-CoV-2 diagnosis (positive PCR) till the time point of study recruitment (C + V + vs. children p = 0.0253, unpaired two tailed unpaired t-test)

*^3^ Months from last vaccination up to study recruitment for adults, follow up time for children (C + V + vs. children p = 0.1172, unpaired two tailed unpaired t-test)

### Preparation of PBMCs and measurement of SARS-CoV-2 reactive T cells

As previously described [29, 30], the cryopreserved PBMCs obtained from the participants were thawed by incubating cryovials 2–3 min at 37 °C in bead bath, washed twice in 37 °C and incubated overnight at 37 °C. 2.5 × 10^6^ PBMCs were plated in 96-U-Well plates in RPMI 1640 media (Life Technologies). The proteins were dissolved per manufacturer’s directions and used at concentration of 1 µg/mL. Each well was stimulated with one of the following SARS-CoV-2 proteins: the pool of B1.617.2 (delta) Spike mutant peptides (Miltenyi Biotec), their reference pool of peptides (Dref) (Miltenyi Biotec), the pool of B.1.1529 (omicron) Spike mutant peptides (Miltenyi Biotec), their reference pool of peptides (Oref) (Miltenyi Biotec) or the complete sequence of WT S-protein (Miltenyi Biotec) or left untreated as a control for 16 h. As a positive control, cells were stimulated with staphylococcal enterotoxin B (1 µg/mL, Sigma-Aldrich). After 2 h, brefeldin A (1 µg/mL, Sigma-Aldrich) was added. Detailed listing of the antibody panels for general phenotyping and T cell activation ex vivo is in Table S1. The PBMCs stimulated overnight were stained with antibodies for 10 min at room temperature in the dark. Stained cells were washed twice with PBS/BSA before preparation for intracellular staining using the Intracellular Fixation & Permeabilization Buffer Set (Thermo Fisher Scientific, USA) per manufacturer’s instructions. Fixed and permeable cells were stained for 30 min at room temperature in the dark with optimal dilution of antibodies against intracellular antigens. All samples were immediately acquired on a CytoFlex flow cytometer (Beckman Coulter, USA). SARS-CoV-2 Spike-reactive CD4 + and CD8 + T cells are defined as CD4 + CD154 + CD137 + and CD8 + CD137 + cells, respectively. Gating strategy is presented in figure S1. Antigen-reactive responses were considered positive after the non-reactive background was subtracted, and more than 0.01% were detectable. No modification to the compensation matrices was required throughout the study. Negative values and values below a threshold of 0.01% were set to zero.

### SARS-CoV-2 neutralizing antibodies

As previously described [30], neutralizing antibodies were analyzed using a propagation-incompetent VSV*DG (firefly luciferase) pseudovirus system bearing SARS-CoV-2 S-protein (wildtype, alpha, delta or omicron) in the envelope. Serial dilutions of serum samples were diluted with the pseudovirus system prior to the infection of Vero E6 cells (kindly provided by Christian Drosten/Marcel Müller Charite, Berlin, Germany) employing pseudovirus. After 18 h incubation in Dulbecco’s minimal essential medium (supplemented with 10% FCS and non-essential amino acids, all Life Technologies, Zug, Switzerland) the firefly luciferase reporter activity was determined. The 50% neutralization dose was determined as the reciprocal antibody dilution causing 50% inhibition of the calculated luciferase reporter.

### Statistics

Flow cytometry data was analyzed using FlowJo version 10.7.1 (BD Biosciences, USA). Statistical analysis was performed using GraphPad Prism v7. Categorical variables are summarized as numbers and frequencies; quantitative variables are reported as median and interquartile range. Normality tests were performed with Shapiro-Wilk test. All applied statistical tests are two-sided. Normal distributed variables were compared with unpaired T test, while not normal distributed variables were compared with unpaired two-tailed Mann-Whitney U and Kruskal-Wallis test. Gender was analyzed with Fisher´s exact test. P values below 0.050 were considered significant; only significant p values are reported in the figures.

## Results

### Characterization of the study groups

Our study group comprised 32 convalescent COVID-19 children (further referred as children) and 34 convalescent COVID-19 adults. 27 out of 34 convalescent COVID-19 adults were at least once vaccinated against SARS-CoV-2 and are further referred as C + V+. 7 out 34 convalescent COVID-19 adults were not vaccinated and are further referred as C + V-. All study participants had a negative SARS-CoV-2 nasal swab tested via RT-PCR on recruitment.

During the acute phase of COVID-19 disease 100% (n = 32) and 93% (n = 25) of the children and C + V+, respectively, presented mild/asymptomatic COVID-19 disease severity without need for hospitalization. The COVID-19 disease severity classification system of the WHO was applied to define the disease severity of the study participants. Of note is, that the time point of SARS-CoV-2 diagnosis in 53% (n = 17) of children was unclear due to asymptomatic COVID-19 disease and could be diagnosed only by serological tests (detection of Spike SARS-CoV-2 IgG, Euroimmun Kit, Lübeck, Germany). However, as the collection of the samples of those subjects took place between April 2021 and August 2021, the calculated maximal follow up time was between 12 and 16 months for those subjects. Only seropositive children were included in the study. The median COVID-19 follow up time was 6 months for children and 12 months for C + V+ (children range 1–16 months, C + V + range 7–16 months). All children participated did not receive a COVID-19 vaccine, whereas 70% (n = 19) of the adults received one COVID-19 vaccine and 30% (n = 8) 2 vaccines at a median time of 5 months (range 4–8) from the timepoint of the recruitment.

The median age of the children study group was 11 years (range 1–18 years), whereas the adult cohort was as expected significantly older, with a median age of 47 years (range 19–68 years, p < 0.0001 two tailed unpaired t test). The children and adult cohorts comprised 37.5% (n = 12) and 63% (n = 17) female participants, respectively and showed no significant gender difference (Fisher´s exact test, p = 0.0692). The demographic and clinical characteristics of the study cohorts are presented in Table 1.

### Significant loss of cross-reactive neutralizing capacity following the viral evolution

**Fig. 1:**
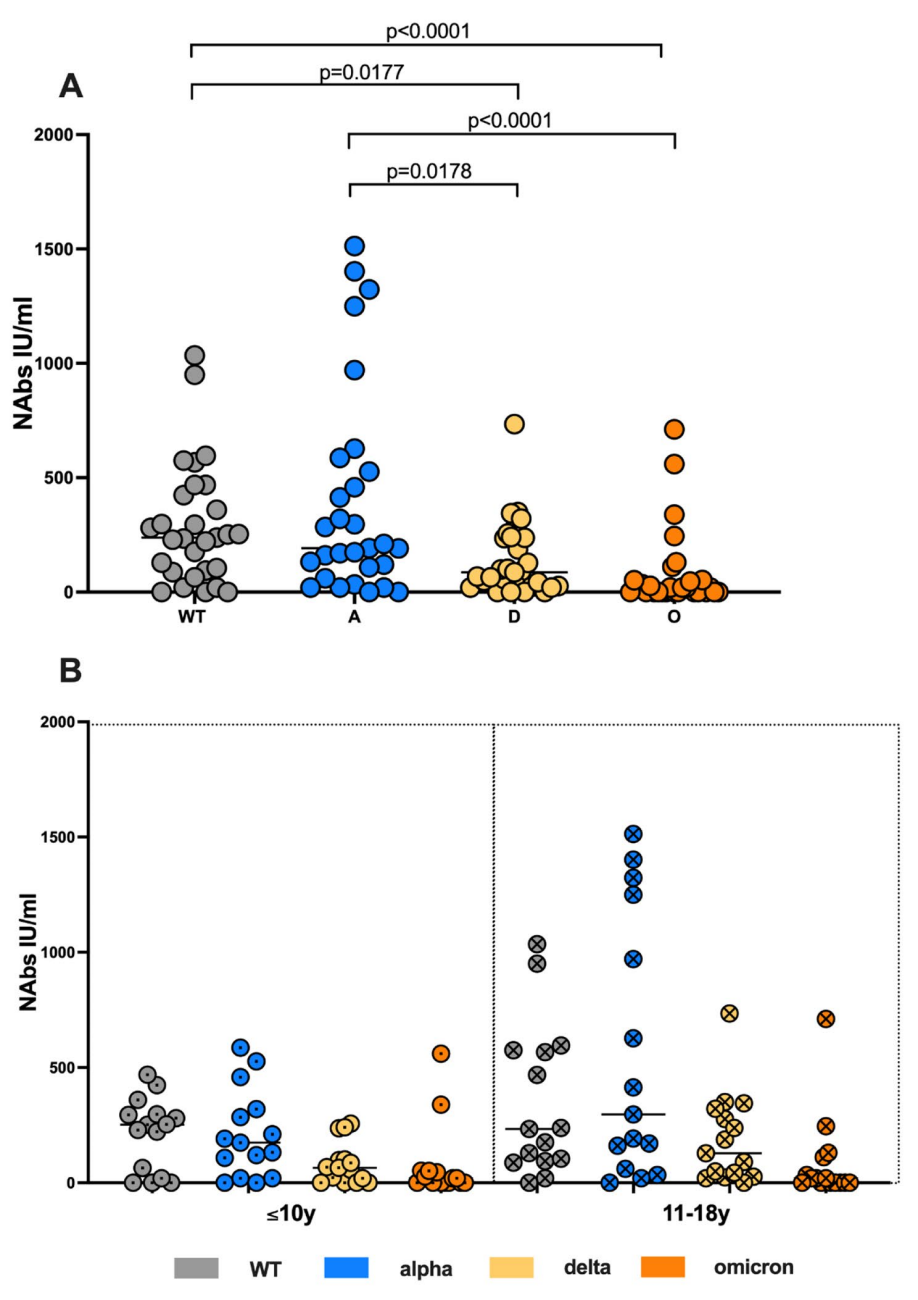
Significant loss of cross-reactive neutralizing capacity against VOC among children 6 months after primary infection. Analysis of WT, alpha, delta and omicron NAbs titers via pseudovirus neutralization propagation-incompetent VSV*DG (firefly luciferase) pseudovirus system bearing SARS-CoV-2 S-protein (wildtype, alpha, delta or omicron) in the envelope among pediatric subjects.***A****) WT-, alpha-, delta- and omicron-NAb.****B****) NAb subanalysis of the pediatric subjects according to age a) ≤10 years old (n = 17) and b)11–18 years old (n = 15). Scatterplots show line at median. Unpaired data were compared with Mann-Whitney-test. P < 0.05 was considered significant, only significant p values are documented in the figures*

First, we addressed the SARS-CoV-2 humoral immunity by measuring the neutralizing antibody titers against the WT, and three influential VOC: alpha, delta and omicron among the convalescent children. Alpha and WT NAbs titers were significantly higher compared to the delta and omicron VOC (Kruskal-Wallis test, Fig. 1A). To assess quantitatively the loss of cross-reactive neutralizing capacity following the viral evolution, we calculated the median NAbs titers ratios, which are the following: delta/WT 34% (range 0-154), omicron/WT 7% (range 0-156%). We observed overall 66% and 93% loss of cross-reactive neutralizing potential against the delta and omicron VOC respectively among the children cohort in a median time of 6 months after the primary infection (fig. S2).

Age-specific variation in immune composition across pediatric age groups have been demonstrated out [31] and in the frame of SARS-CoV-2 infection [32–34]. Therefore, in order to evaluate age-related differences in humoral immune responses to SARS-CoV-2 among the paediatric population, we compared levels of SARS-CoV-2–specific antibodies in children younger than 10yrs old and 11-18yrs old: a) ≤10 years old (n = 17) and b)11–18 years old (n = 15). We found comparable titers of NAb among the two pediatric subgroups (Fig. 1B).

### COVID-19 convalescent children generate SARS-CoV-2 reactive CD4 + and CD8 + T cells cross-recognizing delta and omicron VOCs

**Fig. 2:**
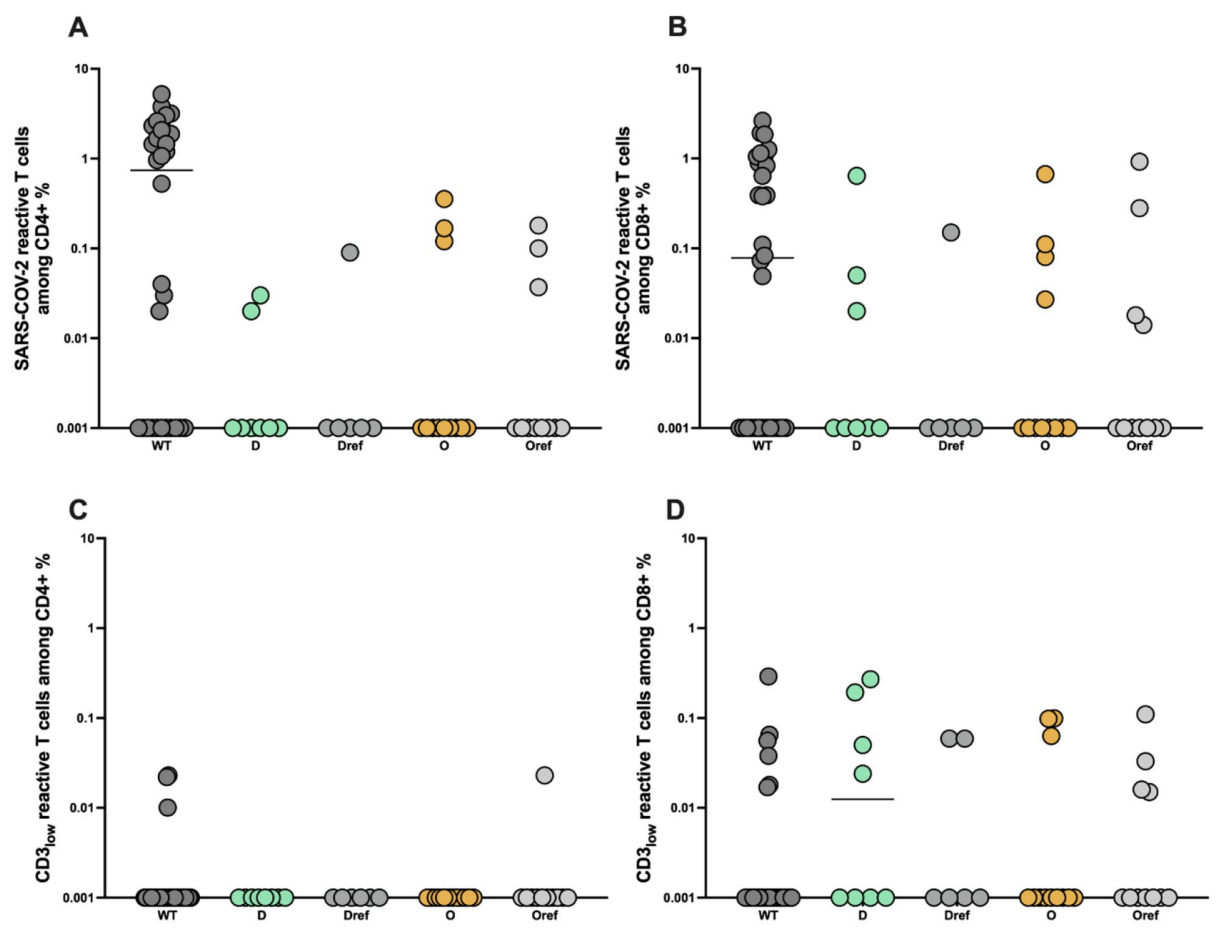
COVID-19 convalescent children can generate SARS-CoV-2 reactive CD4 + and CD8 + T cells with a broad cross-recognition potential. *Characterization of SARSCoV-2 Spike-reactive T cells in pediatric subjects. Blood samples of 32 children were stimulated with one of the following SARS-CoV-2 proteins: the pool of B1.617.2 (delta, D) Spike mutant peptides, their reference pool of peptides (Dref), the pool of B.1.1529 (omicron, O) Spike mutant peptides, their reference pool of peptides (Oref) or the complete sequence of WT S-protein and analyzed by flow cytometry. (****A****) Frequencies of WT-, delta- and omicron-reactive CD4 + T cells. (****B****) Frequencies of WT-, delta- and omicron-reactive CD8 + T cells. (****C****) Avidity of SARS-CoV-2 Spike-reactive T cells as defined by determining the CD3*_*low*_ *+ cells among CD4 + CD154 + CD137 + and (****D****) CD8 + CD137 + cells. Frequencies of WT-, delta- and omicron-reactive CD4 + CD3*_*low*_ *+ T cells are depicted. SARS-CoV-2 Spike-reactive CD4 + and CD8 + T cells are defined as CD4 + CD154 + CD137 + and CD8 + CD137 + cells, respectively. Antigen-reactive responses were considered positive after the non-reactive background was subtracted, and more than 0.01% were detectable. Scatterplots show line at median. Unpaired data were compared with Mann-Whitney-test. P < 0.05 was considered significant, only significant p values are documented in the figures*

Previous data performed in adults demonstrated a sustainable T cell response despite the loss of humoral immunity. Therefore, we analyzed the cellular immune response directed against ancestral wild type (WT) Spike-protein derived from SARS-CoV-2 strain as well as from B1.617.2 (delta) and B.1.1529 (omicron) VOC strains in our convalescent children cohort. For this, we stimulated PBMC obtained from convalescent children with corresponding overlapping peptide pools (Miltenyi Biotec) overnight. As expected, a sustained CD4 + and CD8 + T cell response against WT Spike protein could be detected (Fig. 2A and B). Interestingly and in contrast to antibody data, CD4+ & CD8 + T cells reactive against WT, delta and omicron could be detected with the magnitude comparable between strains (Mann Whitney test, p > 0.05) (Fig. 2A and B).

Bacher et al. suggested the important role of functional avidity for viral clearance especially in context of SARS-CoV-2 infection [35]. Therefore, we also performed an analysis of the avidity of SARS-CoV-2-reactive T cells. Strong TCR activation, which is characteristic of T cells with high TCR avidity, blocks recycling of the TCR-CD3 complex and can be detected by reduced CD3 surface expression, a phenomenon known as high functional avidity [30, 36–38]. Therefore, analyzing the frequencies of CD3_low_ T cells within activated CD4 + or CD8 + T cells will demonstrate the T cells with high avidity, whereas CD3high T cells within activated CD4 + or CD8 + Spike reactive T cells correspond to T cells with a low TCR avidity. Applying this method as performed before [30, 36] (gating strategy, fig. S1), our analysis revealed similar frequencies of Spike-reactive CD4 + and CD8 + CD3_low_ T cells against all studied SARS-CoV-2 strains (Fig. 2C and D). Our results suggest therefore that Spike-reactive CD4 + and CD8 + T cells cross-recognize delta and omicron VOC 6 months after infection in unvaccinated children with equal avidity.

### The titers of neutralizing antibodies in convalescent children are inferior to vaccinated adults but comparable to unvaccinated convalescent adults

**Fig. 3:**
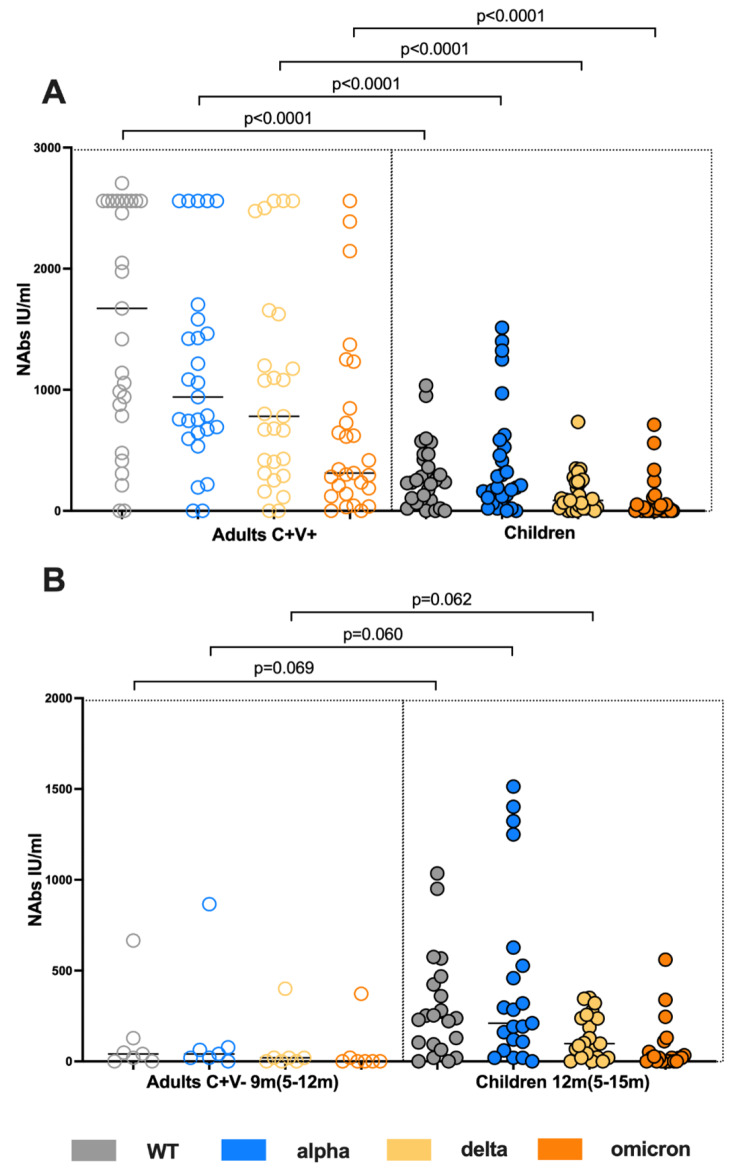
The titers of neutralizing antibodies in convalescent children is inferior to convalescent vaccinated (C + V+) adults but comparable to unvaccinated convalescent (C + V-) adults. *Analysis of WT, alpha, delta and omicron NAbs titers via pseudovirus neutralization assay among pediatric subjects and adults is presented.****A****) NAb comparison among children and C + V+.****B****) NAb comparison between children and C + V-. Scatterplots show line at median. Unpaired data were compared with Mann-Whitney-test. P < 0.05 was considered significant, only significant p values are documented in the figures*

As next, we compared the neutralizing capacity between the children and the C + V+. The C + V + cohort possessed significantly higher titers of neutralizing antibodies compared to the children against all studied strains (Mann Whitney test, WT p < 0.0001, alpha p < 0.0001, delta p < 0.0001, omicron p < 0.0001 (Fig. 3A). The absolute loss of neutralizing capacity against VOC among children as well as adults showed also significant differences (Fisher´s exact test, omicron/WT ratio p = 0.0042, delta/WT ratio p = 0.0003) among the two cohorts (fig. S2).

To explore whether the observed differences are attributed to previous SARS-CoV-2 vaccination among the C + V + cohort, or they represent a more robust humoral response against the virus among adults, we also measured the neutralizing capacity of 7 unvaccinated convalescent adults in median follow up time of 9 months (further referred as C + V-). 20 (out of the 32) children with a median follow up time of 12 months were used for the comparison (Fig. 3B). Comparing these follow up matched cohorts we found that children generated higher titers of neutralizing antibodies against WT, alpha and delta strains however without reaching a statistical significance (Mann Whitney test, WT p = 0.069, alpha p = 0.060, delta 0.062, omicron p = 0.269).

### Higher frequencies of SARS-CoV-2-reactive CD4 + and CD8 + T cells among children compared to adults, characterized by comparable functional avidity

**Fig. 4:**
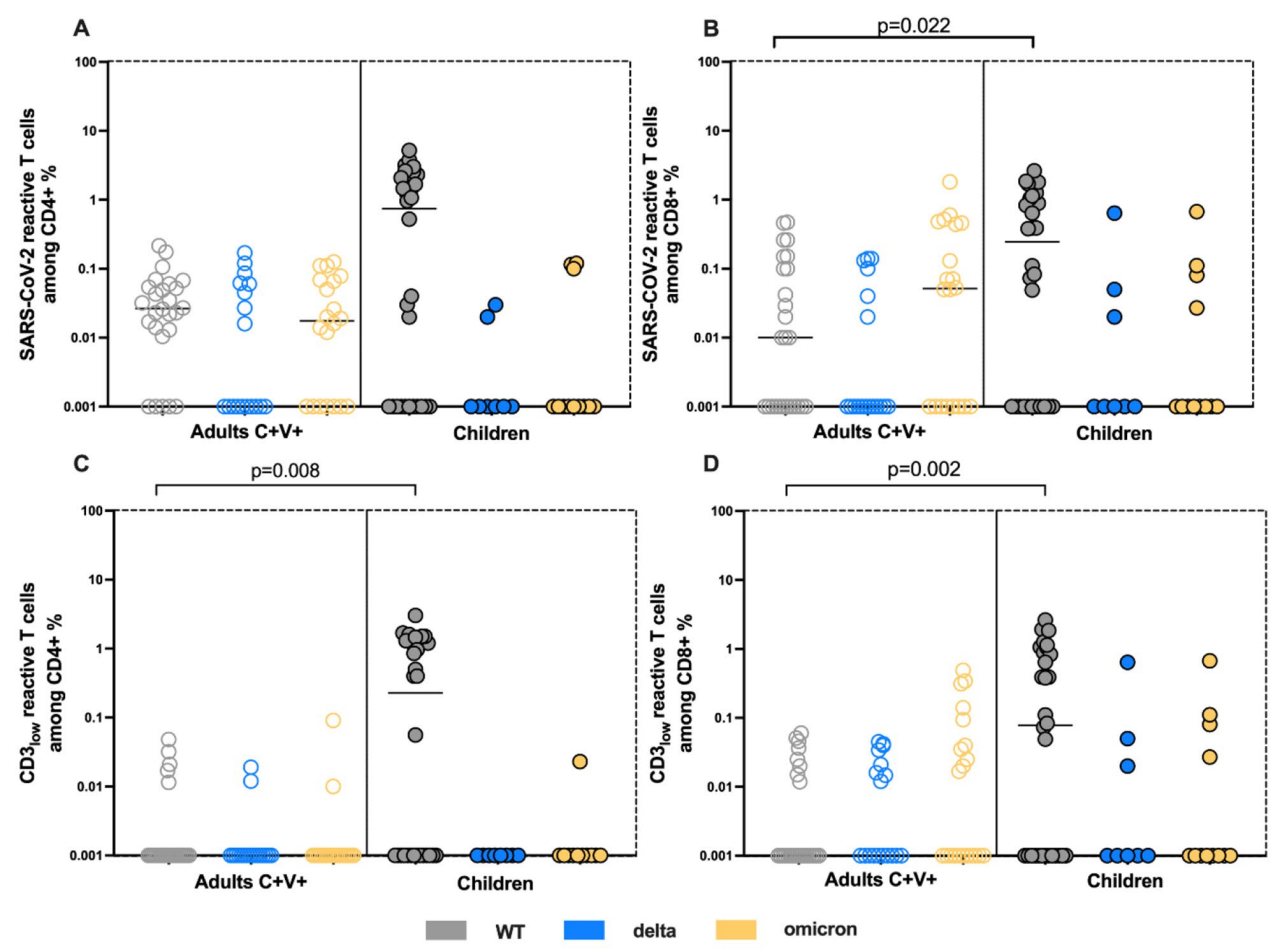
Higher frequencies of SARS-CoV-2-reactive high avidity CD4 + and CD8 + T cells in children compared to adults. *Comparison of SARSCoV-2 S-reactive T cells in children and C + V + subjects is demonstrated. Blood samples of 32 children and 27 C + V + were stimulated with SARS-CoV-2 peptides and analyzed by flow cytometry. (****A****) Frequencies of WT-, delta- and omicron-reactive CD4 + T cells among children and C + V+. (****B****) Frequencies of WT-, delta- and omicron-reactive CD8 + T cells. (****C****) Frequencies of WT-, delta- and omicron-reactive CD4 + CD3*_*low*_ *+ T cells. (****D****) Frequencies of WT-, delta- and omicron-reactive CD8 + CD3*_*low*_ *+ T cells. SARS-CoV-2 S-reactive CD4 + and CD8 + T cells are defined as CD4 + CD154 + CD137 + and CD8 + CD137 + cells respectively. Antigen-reactive responses were considered positive after the non-reactive background was subtracted, and more than 0.01% were detectable. Scatterplots show line at median. Unpaired data were compared with Mann-Whitney-test. P < 0.05 was considered significant, only significant p values are documented in the figures*

Further, we compared SARS-CoV-2 T cell response against WT, delta and omicron between children and adults. The analysis of the total numbers of patients with positive and negative reactive CD4 + and CD8 + T cells showed no significant differences between adults and children (Fisher´s exact test reactive CD4 + T cells adults vs. children WT p = 0.2357, delta p = 0.4048, omicron p = 0.0659) (Fisher´s exact test reactive CD8 + adults vs. children WT p = 1, delta p = 1, omicron p = 0.2734) (Fig. 4A and B). These results indicate that children´s ability to generate reactive T cell response is not inferior compared to adults.

With respect to the magnitude of the cellular immunity, we unexpectedly found higher frequencies of WT-reactive CD4 + and CD8 + T cells in children compared to adults, with WT-reactive CD8 + T cells reaching a statistical significance (Mann-Whitney test, p = 0.008) (Fig. 4A and B). The frequencies of delta- and omicron-reactive CD4 + and CD8 + T cells where similar between the groups (Fig. 4A and B). As next, we addressed the avidity by detecting the CD3_low_ subsets among the antigen-reactive CD4 + and CD8 + T cells. The frequencies of WT-reactive CD4 + and CD8 + CD3_low_ T cells were significantly higher in children compared to adults (Mann-Whitney test, p = 0.022 and p = 0.002 respectively) suggesting a higher avidity of CD4 + and CD8 + T cells directed against WT in children (Fig. 4C and D). Delta- and omicron-reactive CD4 + CD3_low_ and CD8 + CD_3low_ T cells showed similar frequencies in children and adults (Fig. 4C and D).

Lastly, we compared the frequencies of SARS-CoV-2- reactive T cells of the children cohort with the C + V- adult cohort. The frequencies of WT-, delta- and omicron-reactive CD4 + T cells were comparable (fig. S3A) between the two cohorts, as well as the frequencies of delta and omicron CD4 + CD3_low_ T cells (fig. S3B). We found significantly higher frequencies of WT-reactive CD4 + CD3_low_ T cells in children compared to C + V- (fig. S3B) (Mann-Whitney test, p = 0.046). Regarding the CD8 + reactive T cells, frequencies of WT-, delta- and omicron-reactive CD8 + T cells were comparable between the two cohorts (fig. S3C) (Mann-Whitney test, p = 0.08). CD8 + CD3_low_ T cell frequencies were also similar for both cohorts (fig. S3D).

### WT-reactive CD4 + and CD8 + CD3_low_ T cells among children are functional, with the ability of prominent cytokine production

Cytokine production and polyfunctionality of reactive T cells have been described as a hallmark of protective immunity in viral infections [39–41]. Addressing this point, we analyzed the IFN-γ, TNF-α and IL-2 cytokines, as well as effector molecule GrB expression among WT-reactive CD4 + CD3_low_ and CD8 + CD3_low_ T cells. In children, WT-reactive CD4 + CD3_low_ T cells producing IL2 and TNFα showed significantly higher frequencies compared to GrB and IFNγ producing WT-reactive CD4 + CD3_low_ T cells (Mann-Whitney test, p = 0.0002 and p < 0.0001 respectively) (fig. S4A). Adults showed similar frequencies of WT-reactive CD4 + CD3_low_ T cells, except for the IFNγ producing WT-reactive CD4 + CD3_low_ T cells, which showed significantly higher frequencies compared to GrB and TNFα producing WT-reactive CD4 + CD3_low_ T cells (Mann-Whitney test p = 0.0002 and p = 0.022 respectively) (fig. S4A). Direct comparison of the WT-reactive CD4 + CD3_low_ T cells between the two study groups revealed significantly higher frequencies of IFNγ producing WT-reactive CD4 + CD3_low_ T cells in adults compared to children, but comparable frequencies of GrB, IL2 and TNFα producing WT-reactive CD4 + CD3_low_ T cells (fig. S4A).

Regarding the WT-reactive CD8 + CD3_low_ T cells, we found high frequencies of IFNγ, IL2, GrB and TNFα producing WT-reactive CD8 + CD3_low_ T cells in children with comparable frequencies (Kruskal-Wallis and Mann-Whitney test) (fig. S4B). Similarly, IFNγ, IL2, GrB and TNFα producing WT-reactive CD8 + CD3_low_ T cells showed prominent but comparable frequencies withih the adult cohort (Kruskal-Wallis and Mann-Whitney test) (fig. S4B). Direct comparison of the WT-reactive CD8 + CD3_low_ T cells between the two study groups revealed overall higher frequencies of cytokine producing WT-reactive CD8 + CD3_low_ T cells in children compared to adults, while IFNγ and TNFα producing WT-reactive CD8 + CD3_low_ T cells showed significantly higher frequencies in children compared adults (Mann Whitney test, p = 0.039 and p = 0.025 respectively) (fig. S4B).

In total, we observed that WT-reactive CD4 + and CD8 + CD3_low_ T cells in children are functional, with the ability of prominent cytokine production with frequencies comparable and partially higher compared to adults.

### Re-analysis of NAb titers and SARS-CoV-2 specific T cell frequencies excluding pediatric subjects with unknown convalescent duration shows no alteration of the results

The time between the SARS-Cov2 infection and blood analysis is crucial, when interpreting humoral and cellular immunity. We repeated, therefore, the statistical analysis excluding the pediatric subjects with unknown follow up time (17 out of 32). WT, delta and omicron reactive CD4 + and CD8 + T cells showed similar frequencies among pediatric patients and comparable functional avidity (fig. S5). WT and alpha NAb titers were significantly higher than omicron NAb titers among children (fig. S6). As demonstrated above, C + V + adults generated significantly higher NAb titers against all variants compared to children (fig. S6). Lastly, frequencies of WT, delta and omicron reactive CD4 + and CD8 + T cells in children, as well as their avidity, are comparable, and slightly higher, to adults (fig. S7). Taken together, the re-analysis excludes, that convalescence duration biases the results.

## Discussion

Although SARS-CoV-2 memory and its cross-recognition potential against VOC among adults in convalescence is widely studied, similar immune profiling among convalescent COVID-19 children is missing. This study offers a detailed immune analysis of the SARS-CoV-2 memory and cross-recognition potential against the most influential VOC among unvaccinated convalescent children as compared to convalescent (vaccinated and unvaccinated) adults. We demonstrate that COVID-19 convalescent children can generate SARS-CoV-2 reactive CD4 + and CD8 + T cells with broad cross-recognition potential against the studied VOC. The detected cross-reactive CD4 + and CD8 + T cell response presented a stronger response among children compared to adults, which were characterized by the higher frequencies, higher avidity and functionality of T cells. Furthermore, unvaccinated children have neutralizing capacity comparable to unvaccinated adults. However, due to the current immune dynamics and differentiated vaccination rates, unvaccinated children possess an inferior SARS-CoV-2 neutralizing capacity compared to adults.

Although some previous studies showed lower SARS-CoV-2 specific T cell responses among children compared to adults one month and 4 months post-infection [15, 42], our data are in agreement with some recent studies demonstrating a sustained cross-reactive spike specific T cell responses compared to adults, independent of the convalescence duration [32, 43].

The main scientific attention is currently drawn, to how viral evolution and SARS-CoV-2 mutation affect immune dynamics among adults. Ours [44] and emerging independent data [45–52] show that despite the apparent loss of neutralization potential due to viral evolution, SARS-CoV-2 reactive T cell response is preserved with potential for cross-recognition of different VOC among adults. Paul et al. reported significant cross-reactivity of CD4 + T cell responses to the beta variant among children after natural infection, however similar data regarding further VOC among the pediatric population is missing. Here, we demonstrate that Spike-reactive T cells from unvaccinated convalescent children can cross-recognize delta and omicron VOC with a magnitude and avidity comparable to vaccinated convalescent adults. Our study also assessed the SARS-CoV-2 humoral immunity in children. It has been proven, that asymptomatic or mild symptomatic SARS-CoV-2 infection elicits durable neutralizing antibody responses in children and adolescents (32–33). Our results indicate that the titers of NAb are not significantly differentiated by age in our studied cohorts, and are in line with the literature [33, 43, 53–55].

In the same line with independent studies analyzing adult cohorts (56–57), we report a significant loss of cross-reactive neutralizing capacity against the studied VOC among children 6 months after primary infection. The loss of cross-reactive neutralizing potential among the pediatric subjects was comparable with unvaccinated convalescent adults. However, when the pediatric unvaccinated subjects were compared with convalescent vaccinated adults, we observed as expected a significant inferior pediatric humoral response.

Our study has limitations that should be addressed. First, the time between disease onset and the study recruitment was unknown for 53% of the asymptomatic pediatric subjects. However, as samples were collected between April and August 2021 (between 12 and 16 months after the initial outbreak of COVID-19 in Nord Rhine Westphalia, Germany), we calculated a maximum convalescent duration between 11 and 15 months. Second, SARS-CoV-2 infection among the pediatric (unvaccinated) cohort was based on serological detection of Spike SARS-CoV-2 IgG antibodies due to the absence of PCR screening, while the infecting variants were predicted according to temporal viral spreading. The children cohort included slightly more female than male subjects. Furthermore, HLA type seems to play an important role in reduced reactivity for certain VOC in adults. However, HLA-typing was out of scope of our project. Future studies could report on HLA-typing and its role in pediatric antiviral immunity as it has been proven of significance among adults [58]. Lastly, the cohort of unvaccinated convalescent adults is rather small, which is explained by the successful vaccination program and lack of access to non-vaccinated group.

A key motivation for our study was to provide data on SARS-CoV-2 cellular and humoral immunity that is important for the current vaccination strategies in pediatric population. Taken together, our results contain two key messages:

- unvaccinated convalescent children are able to generate neutralizing antibodies that are at least partially cross-reactive to the newly evolved VOC. However, their titers are inferior as compared to vaccinated convalescent adults.
- despite the inferior humoral immunity, convalescent children seem to generate and maintain Spike-specific T-cells with a broad cross-reactive potential comparable or even superior to convalescent unvaccinated or convalescent vaccinated adults.

### Electronic supplementary material

Below is the link to the electronic supplementary material.

Supplementary Material 1

## Data Availability

The datasets used and/or analysed during the current study available from the corresponding author on reasonable request.
