# Supplementary material for: Fading SARS-CoV-2 humoral VOC cross-reactivity and sustained cellular immunity in convalescent children and adolescents
Source: BMC Infect Dis. 2023 Nov 22;23:818. doi: 10.1186/s12879-023-08805-9 (PMC10664582; doi:10.1186/s12879-023-08805-9)
Supplement: Supplementary file 1 — Supplementary Material 1 [file 12879_2023_8805_MOESM1_ESM.docx]

**Fading SARS-CoV-2 humoral VOC cross-reactivity versus sustained cellular immunity in convalescent children and adolescents**

**Supplemental Material - Table of Contents**

**Supplementary Table**

**Table S1:** Fluorochrome coupled antibodies and fluorescent dye for analysis of SARS-CoV-2 reactive T cells

**Table S2.** Detailed results of the pseudovirus assay

**Supplementary Figure**

**Figure S1.** Flow cytometry gating strategy for identification and quantification of SARS-CoV-2 reactive T cells

**Figure S2.** Neutralizing capacity and absolute loss of neutralizing capacity against VOC

**Figure S3.** Higher frequencies of SARS-CoV-2-reactive high avidity CD4+ and CD8+ T cells in children compared to adults (C+V- included)

**Figure S4.** Functional WT-reactive CD4+ and CD8+CD3_low_ T cells among children with the ability of prominent cytokine production.

**Figure S5.** Analysis of SARS-CoV-2 specific T cell frequencies in pediatric subjects with known convalescent duration

**Figure S6.** Analysis of NAb titers in pediatric subjects with known convalescent duration

**Figure S7.** Comparison of SARS‑CoV-2 S-reactive T cells between C+V+ and children in subjects with known convalescent duration

**Table S1:** Fluorochrome coupled antibodies and fluorescent dye for analysis of SARS-CoV-2 reactive T cells

| **Antibodies or fluorescent dye** | **Fluorochrome** | **Source** | **Cat. Nr.** |
| --- | --- | --- | --- |
| Fixable Viability-Dye | eFluor780 | eBioscience | 65-0865-14 |
| anti CD4 (clone OKT4) | A700 | BioLegend | 317426 |
| anti CD8 (clone RPA-T8) | V500 | BD Biosciences | 560775 |
| anti CD137 (4-1BB) (clone 4B4-1) | PE-Cy7 | BioLegend | 309818 |
| anti CD154 (CD40L) (clone 24-31) | A647 | BioLegend | 310818 |
| anti CD3 (clone OKT3) | BV785 | BioLegend | 317330 |

**Table S2.** Detailed results of the pseudovirus assay

| **Patient ID** | **WT** | **Alpha** | **Delta** | **Omicron** |
| --- | --- | --- | --- | --- |
| 04-KindCoV1 | 0 | 0 | 0 | 0 |
| 04-KindCoV7 | 87.34 | 33.57 | 26.7 | 0 |
| 04-KIndCoV9 | 20 | 20 | 20 | 0 |
| 04-KindCoV23 | 94.17 | 160.7 | 90.48 | 20 |
| 04-KindCoV30 | 238.3 | 60.38 | 349.8 | 33.86 |
| 04-KindCoV31 | 0 | 0 | 0 | 0 |
| 04-KindCoV33 | 294.4 | 131.6 | 56.81 | 0 |
| 04-KindCoV35 | 567.1 | 296.4 | 187.1 | 20 |
| 04-KindCoV38 | 233.7 | 413.7 | 23.6 | 0 |
| 04-KindCoV45 | 175.9 | 170.1 | 49.48 | 0 |
| 04-KindCoV51 | 423.9 | 191 | 22.81 | 0 |
| 04-KindCoV52 | 0 | 20 | 0 | 0 |
| 04-KindCoV53 | 20 | 20 | 0 | 0 |
| 04-KindCoV55 | 0 | 0 | 0 | 0 |
| 04-KindCoV56 | 64.26 | 120 | 98.7 | 20 |
| 04-KindCoV58 | 575.1 | 1402 | 276.3 | 110.9 |
| 04-KindCoV69 | 950.4 | 1250 | 345 | 245.3 |
| 04-KindCoV71 | 104.6 | 191.7 | 127.7 | 20 |
| 04-KindCoV72 | 251.9 | 526.4 | 101.4 | 52.65 |
| 04-KindCoV74 | 1035 | 1323 | 321 | 130 |
| 04-KindCoV75 | 280.1 | 458.1 | 67.67 | 20 |
| 04-KindCoV76 | 128.6 | 626.9 | 43.59 | 0 |
| 04-KindCoV77 | 468.2 | 1513 | 237.8 | 0 |
| 04-KindCoV78 | 229.3 | 285 | 20 | 0 |
| 04-KindCoV80 | 360 | 319.7 | 236.1 | 560 |
| 04-KindCoV83 | 297.5 | 586.4 | 64.88 | 45.91 |
| 04-KindCoV84 | 221.3 | 210.6 | 256.4 | 338.7 |
| 04-KindCoV94 | 596.1 | 970.4 | 734.5 | 711.1 |
| 04-KindCoV98 | 253.5 | 108.1 | 86.49 | 27.17 |
| 04-KindCoV103 | 468.7 | 173.8 | 240.6 | 51.96 |
| 04-CoV297-V001 | 40.86 | 40.91 | 20 | 0 |
| 04-CoV300-V001 | 1205 | 331.7 | 453.7 | 249 |
| 04-CoV303-V001 | 2560 | 2560 | 2560 | 2560 |
| 04-CoV310-V001 | 0 | 20 | 0 | 0 |
| 04-CoV312-V001 | 2560 | 2560 | 2560 | 2560 |
| 04-CoV315-V001 | 2560 | 2560 | 2560 | 1251 |
| 04-CoV318-V001 | 1418 | 939.2 | 309.6 | 120.4 |
| 04-CoV265-V003 | 2707 | 1582 | 1200 | 1372 |
| 04-CoV319-V001 | 2560 | 1464 | 1174 | 726.1 |
| 04-CoV323-V001 | 2560 | 2081 | 2137 | 1516 |
| 04-CoV511 V001 | 1290 | 1654 | 711.8 | 610.8 |
| 04-CoV512 V001 | 48.97 | 44.29 | 63.9 | 20 |
| 04-CoV513 V001 | 2560 | 2560 | 2560 | 2560 |
| 04-CoV514 V001 | 2560 | 1893 | 655.9 | 2560 |
| 04-CoV518 V001 | 2560 | 2560 | 1508 | 2560 |
| 04-CoV519 V001 | 2018 | 2560 | 978.5 | 285.3 |

**
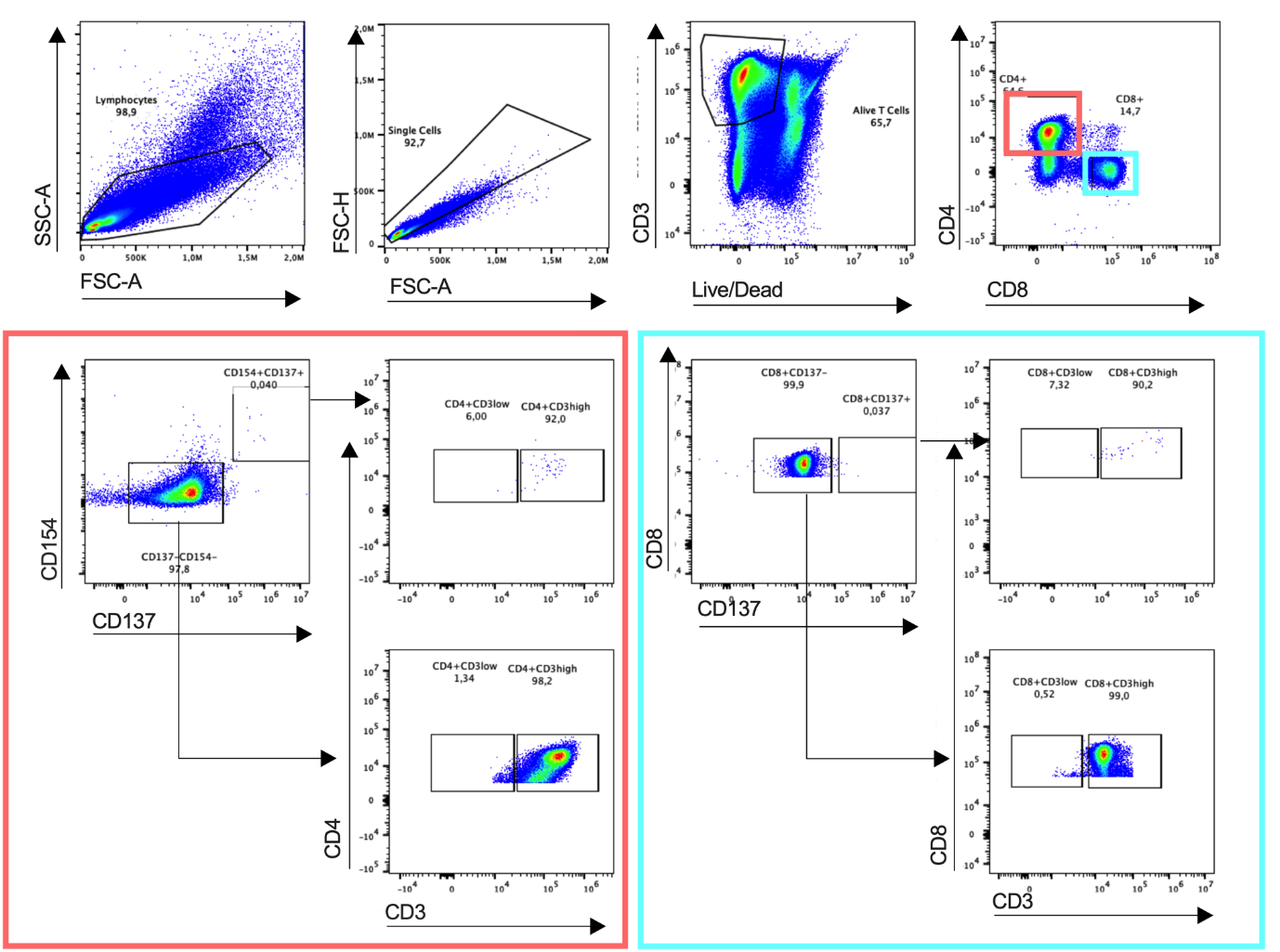
**

**Figure S1. Flow cytometry gating strategy for identification and quantification of SARS-CoV-2 reactive T cells.** PBMCs were stimulated for 16 h with one of the following: the pool of B1.617.2 (delta) Spike mutant peptides (Miltenyi Biotec), their reference pool of peptides (Miltenyi Biotec), the pool of B.1.1529 (omicron) Spike mutant peptides (Miltenyi Biotec), their reference pool of peptides (Miltenyi Biotec), the complete sequence WT S-protein (Miltenyi Biotec) or left untreated as a control. Living single lymphocytes were analyzed for expression of CD3, CD4, and CD8. CD4+ T cells (orange boxes) were analyzed for the expression of CD154 and CD137. CD8+ T cells (blue boxes) were analyzed for expression of CD137. Both CD4+ and CD8+ T cells were further analyzed for the production of cytokines IFNγ, TNFα, IL2 and GrB. Evaluation of the memory subsets was performed using the markers CCR7 and CD45RA (T_CM_=CD45RA-CCR7+, T_NAIVE_=CD45RA+CCR7+, T_EM_=CD45RA-CCR7- T_EMRA_=CD45RA+CCR7-). Furthermore, CD4+CD154+CD137+, CD8+CD137+ and cells were analyzed for the expression of CD3_low_. Representative example of 32 convalescent children and 34 convalescent adults. Plots of a pediatric study subject are depicted.


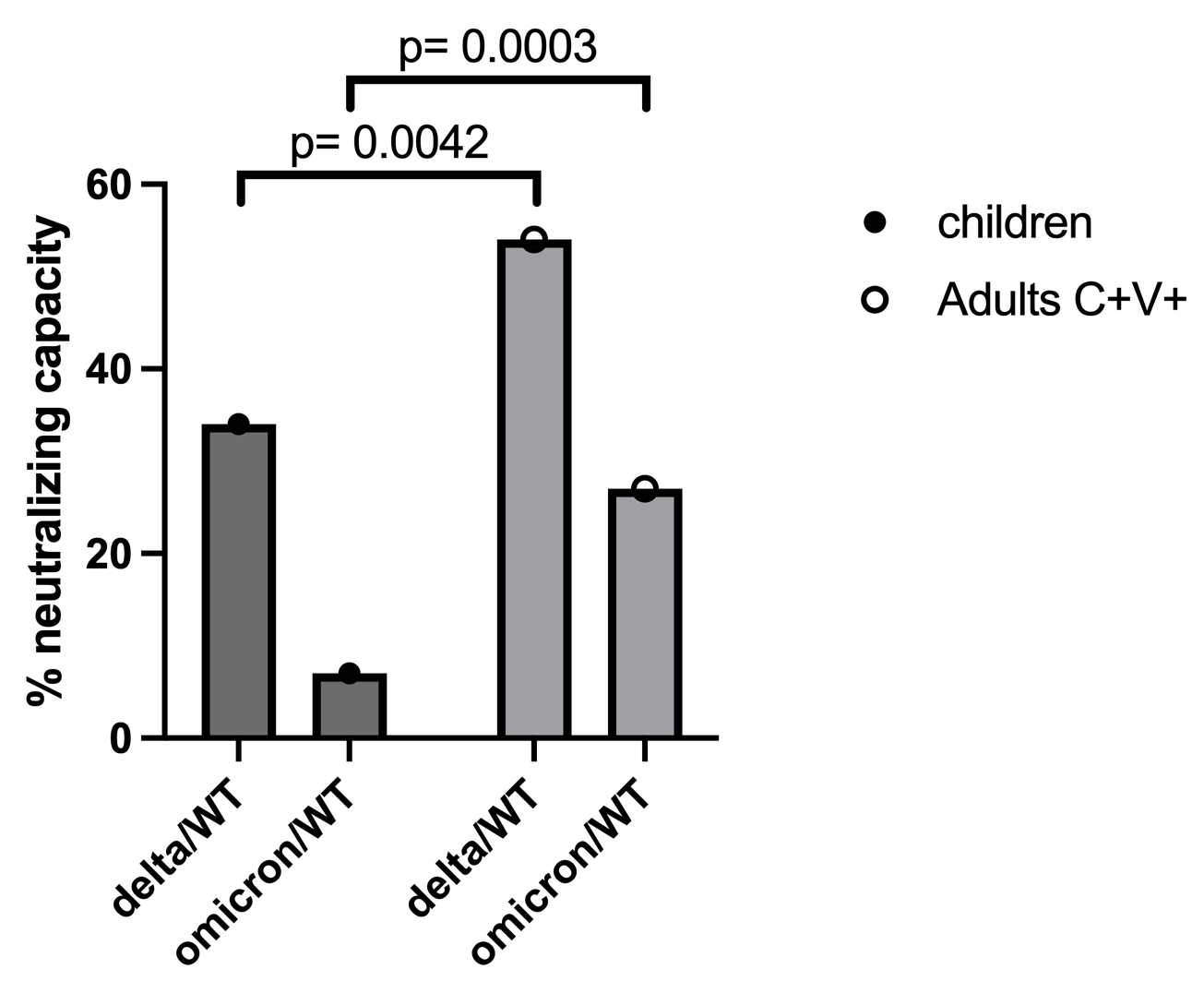


**Figure S2.** **Neutralizing capacity and absolute loss of neutralizing capacity against VOC.** To assess quantitatively the loss of cross-reactive neutralizing capacity following the viral evolution, we calculated the median NAbs titers ratios (delta/WT, omicron/WT). Scatterplots show line at median. Data were compared with exact Fisher test. P<0.05 was considered significant, only significant p values are documented in the figures.


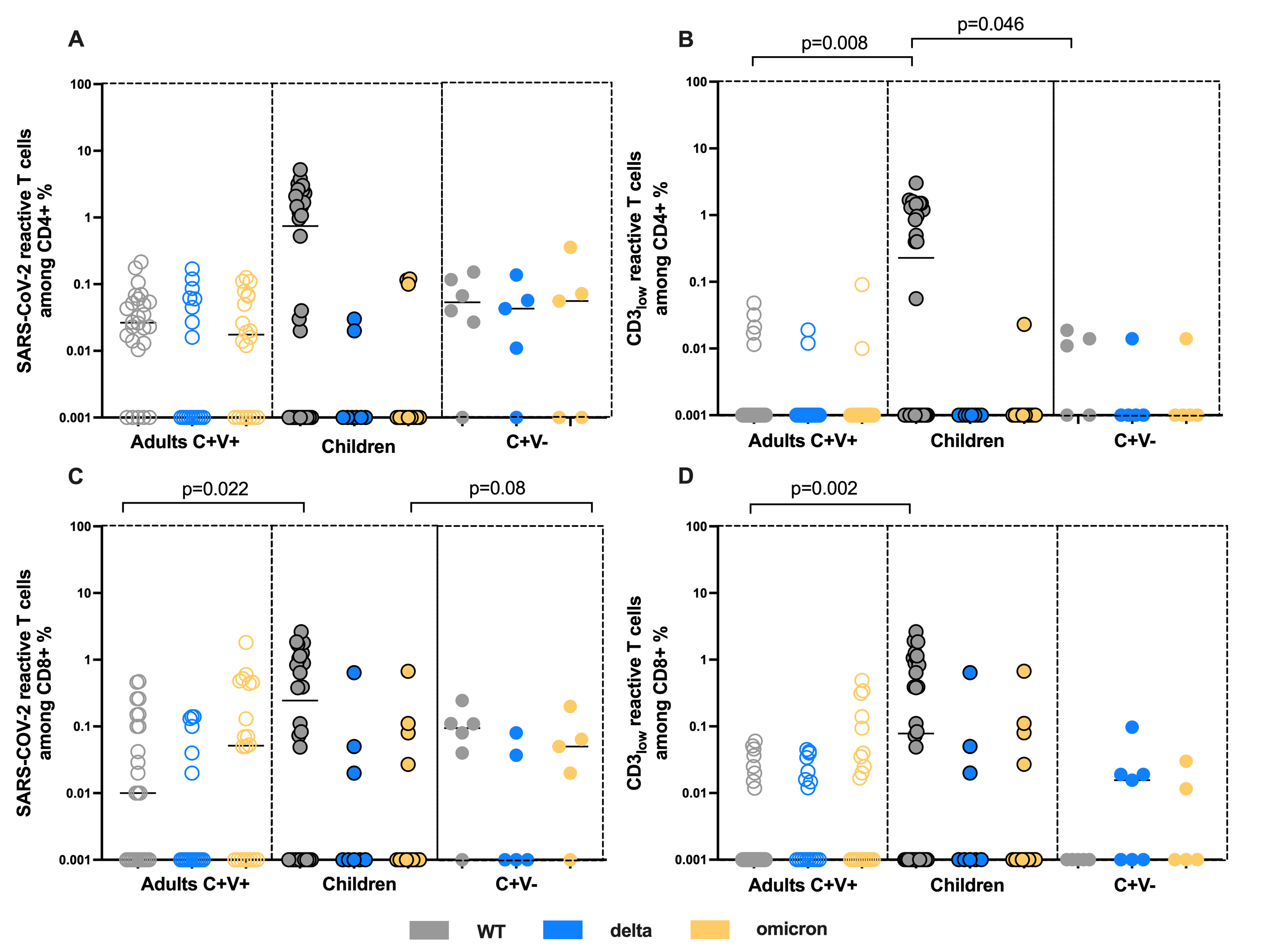


**Figure S3. Higher frequencies of SARS-CoV-2-reactive high avidity CD4+ and CD8+ T cells in children compared to adults (C+V- included).** Comparison of SARS‑CoV-2 S-reactive T cells in children, C+V+ and C+V- subjects is demonstrated. (A) Frequencies of WT-, delta- and omicron-reactive CD4+ T cells. (B) Frequencies of WT-, delta- and omicron-reactive CD4+CD3low+ T cells. (C) Frequencies of WT-, delta- and omicron-reactive CD8+ T cells. (D) Frequencies of WT-, delta- and omicron-reactive CD8+CD3low+ T cells. SARS-CoV-2 S-reactive CD4+ and CD8+ T cells are defined as CD4+CD154+CD137+ and CD8+CD137+ cells respectively. Antigen-reactive responses were considered positive after the non-reactive background was subtracted, and more than 0.01% were detectable. Scatterplots show line at median. Unpaired data were compared with Mann-Whitney-test. P<0.05 was considered significant, only significant p values are documented in the figures.


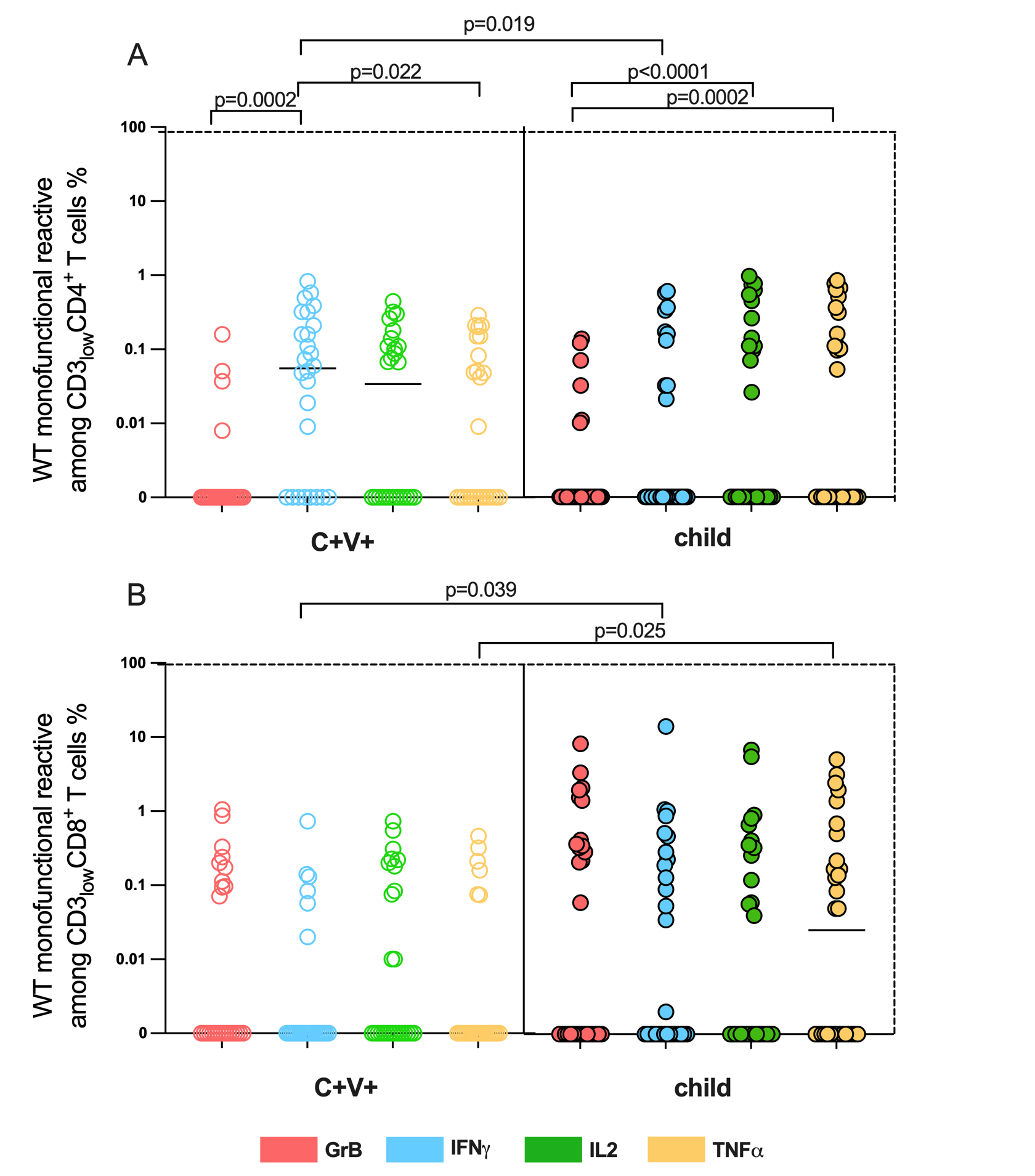


**Figure S4. Functional WT-reactive CD4+ and CD8+CD3_low_ T cells among children with the ability of prominent cytokine production.** The frequencies of IL2, IFNγ, TNFα or GrB producing WT-reactive CD4+ and CD8+CD3_low_ T cells were analyzed among pediatric subjects and C+V+. (A) GrB, IFNγ, IL2 and TNFα producing SARS-CoV-2 reactive CD4+CD3_low_ T cells. (B) GrB, IFNγ, IL2 and TNFα producing SARS-CoV-2 reactive CD8+CD3_low_ T cells. SARS-CoV-2 S-reactive CD4+ and CD8+ T cells are defined as CD4+CD154+CD137+ and CD8+CD137+ cells respectively. Antigen-reactive responses were considered positive after the non-reactive background was subtracted, and more than 0.01% were detectable. Scatterplots show line at median. Unpaired data were compared with Mann-Whitney-test and Kruskal-Wallis test. P<0.05 was considered significant, only significant p values are documented in the figures.

**
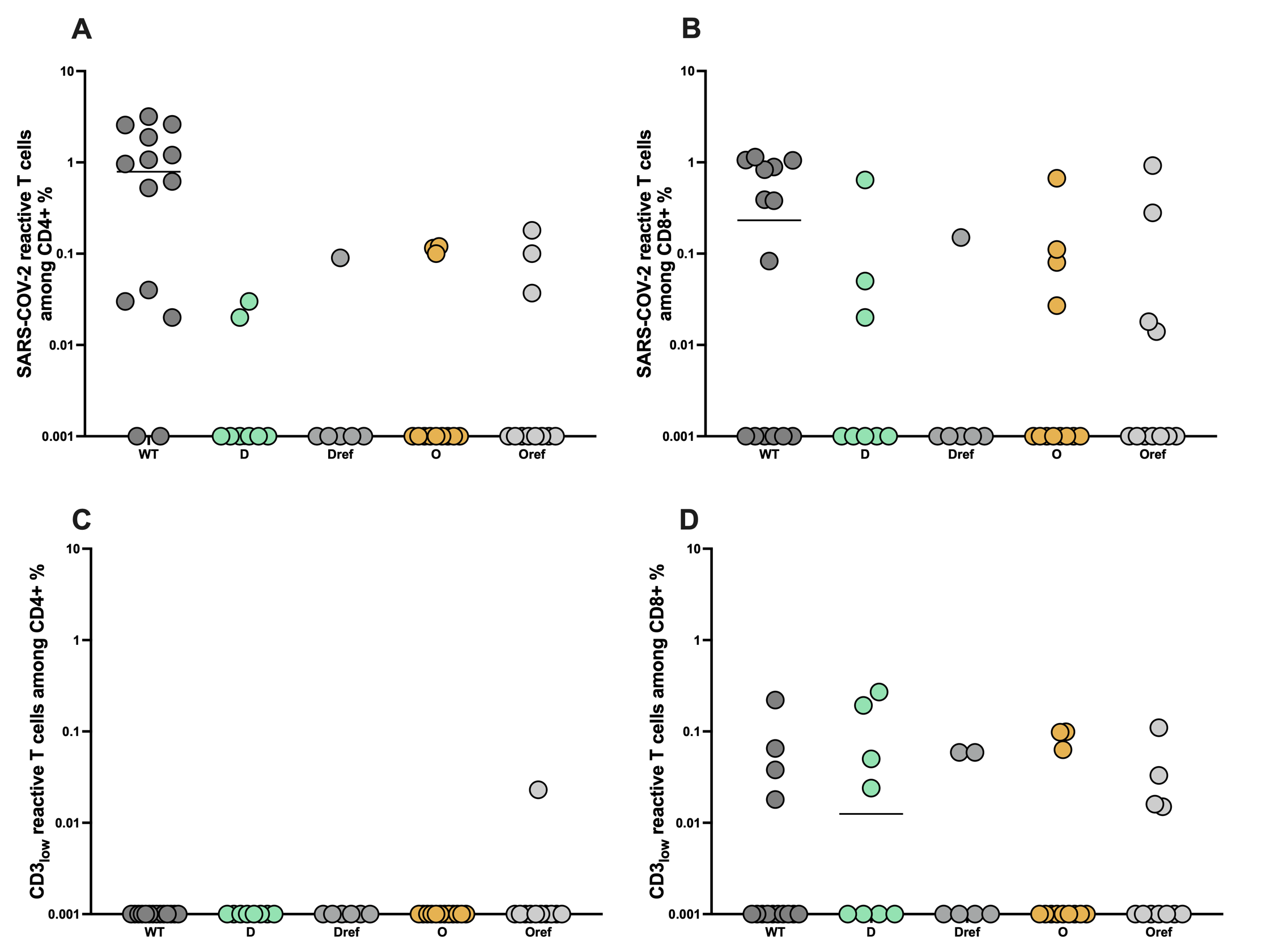
**

**Figure S5.** **Analysis of SARS-CoV-2 specific T cell frequencies in pediatric subjects with known convalescent duration.** *Blood samples were stimulated with one of the following SARS-CoV-2 proteins: the pool of B1.617.2 (delta, D) Spike mutant peptides, their reference pool of peptides (Dref), the pool of B.1.1529 (omicron, O) Spike mutant peptides, their reference pool of peptides (Oref) or the complete sequence of WT S-protein and analyzed by flow cytometry.* Re-analysis of SARS‑CoV-2 Spike-reactive T cells was performed in pediatric subjects excluding patients with unknown convalescent duration. (A) Frequencies of WT-, delta- and omicron-reactive CD4+ T cells. (B) Frequencies of WT-, delta- and omicron-reactive CD8+ T cells.

Avidity of SARS-CoV-2 Spike-reactive T cells as defined by determining the CD3_low_+ cells among (C) CD4+CD154+CD137+ and (D) CD8+CD137+ cells. Frequencies of WT-, delta- and omicron-reactive CD4+CD3_low_+ T cells are depicted. SARS-CoV-2 Spike-reactive CD4+ and CD8+ T cells are defined as CD4+CD154+CD137+ and CD8+CD137+ cells, respectively. Antigen-reactive responses were considered positive after the non-reactive background was subtracted, and more than 0.01% were detectable. Scatterplots show line at median. Unpaired data were compared with Mann-Whitney-test. P<0.05 was considered significant, only significant p values are documented in the figures.

**
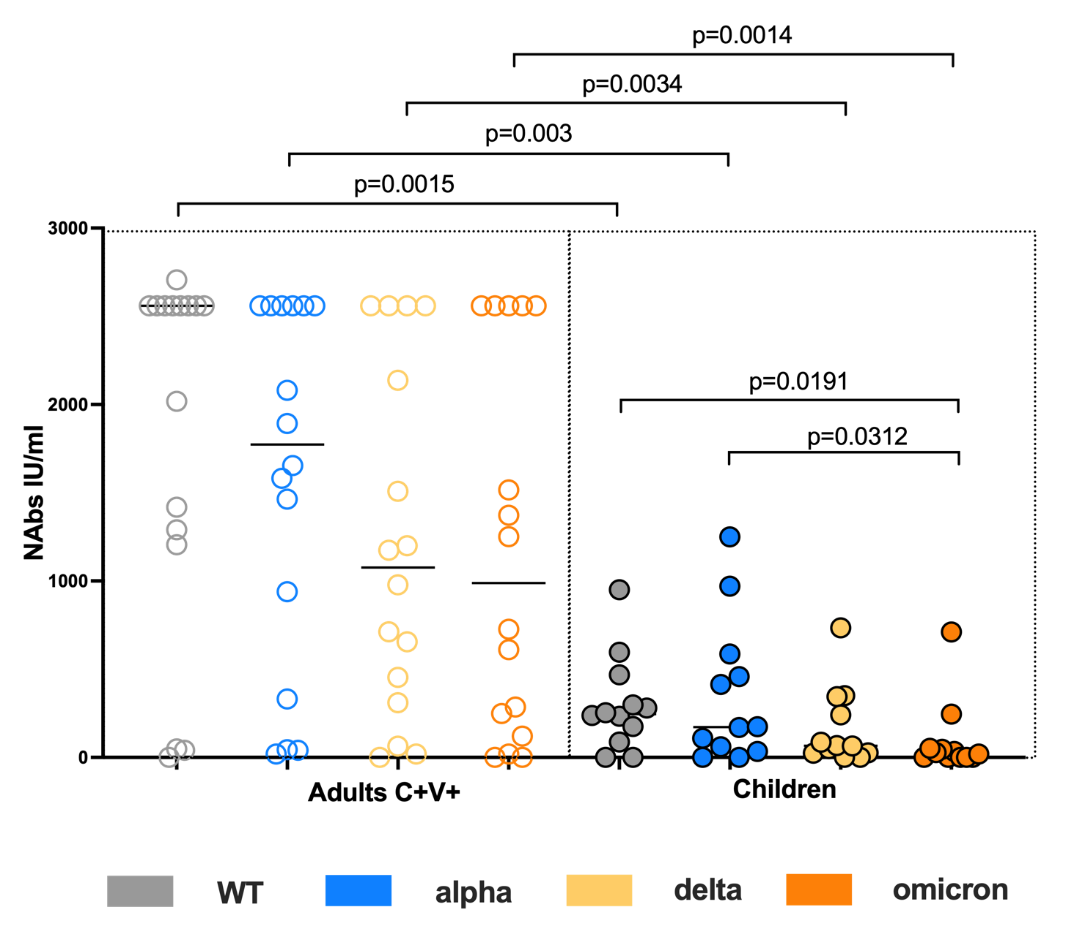
**

**Figure S6.** **Analysis of NAb titers in pediatric subjects with known convalescent duration.** Re-analysis of WT, alpha, delta and omicron NAbs titers via pseudovirus neutralization assay was performed in pediatric patients excluding subjects with unknown convalescent time. Unpaired data were compared with Mann-Whitney-test. P<0.05 was considered significant, only significant p values are documented in the figures.

**
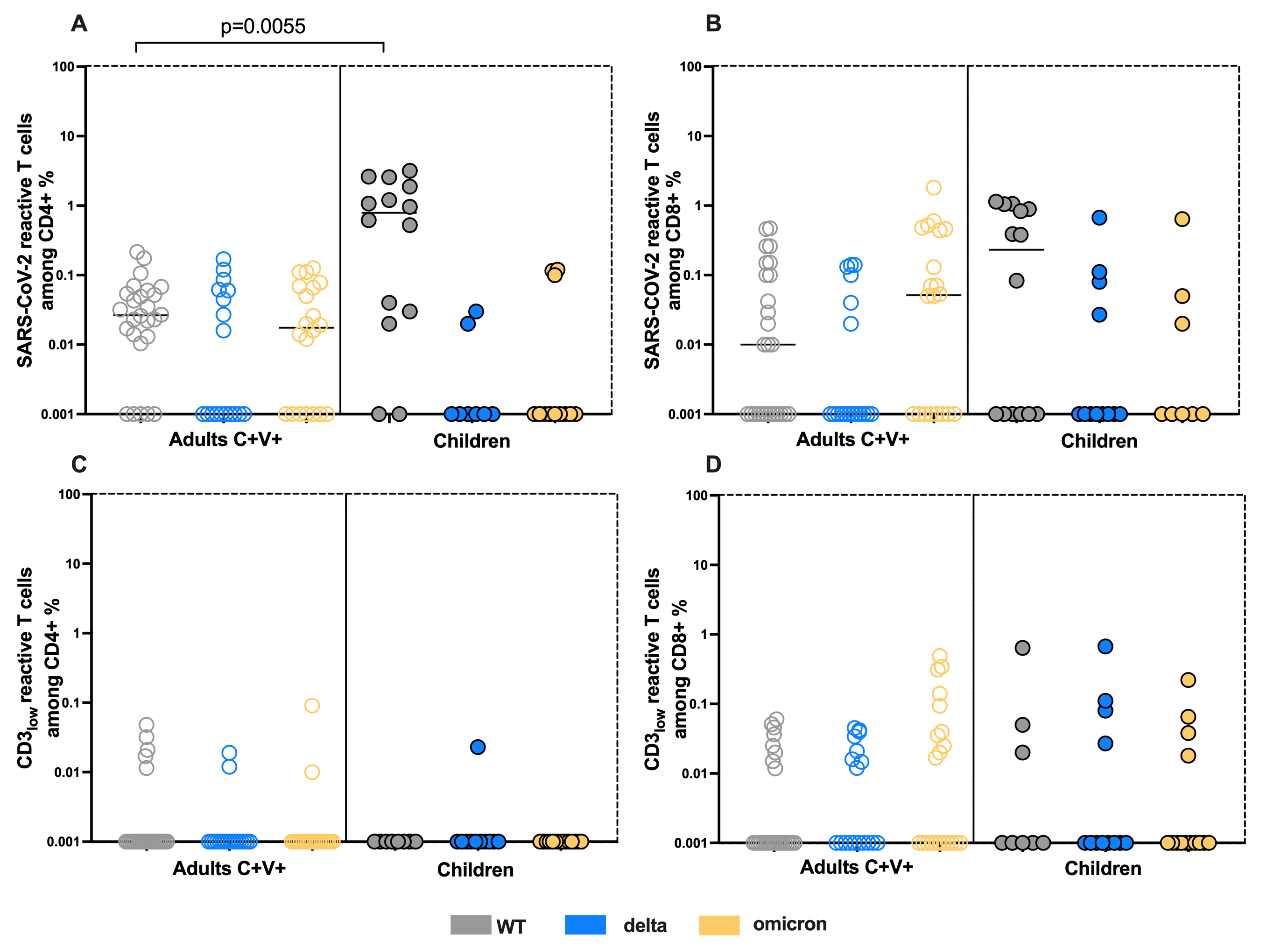
**

**Figure S7. Comparison of SARS‑CoV-2 S-reactive T cells between C+V+ adults and children in subjects with known convalescent duration.** Comparison of SARS‑CoV-2 S-reactive T cells among C+V+ adults and children was performed after subjects with unknown convalescent duration were excluded. (A) Frequencies of WT-, delta- and omicron-reactive CD4+ T cells among children and C+V+. (B) Frequencies of WT-, delta- and omicron-reactive CD8+ T cells. (C) Frequencies of WT-, delta- and omicron-reactive CD4+CD3_low_+ T cells. (D) Frequencies of WT-, delta- and omicron-reactive CD8+CD3_low_+ T cells. SARS-CoV-2 S-reactive CD4+ and CD8+ T cells are defined as CD4+CD154+CD137+ and CD8+CD137+ cells respectively. Antigen-reactive responses were considered positive after the non-reactive background was subtracted, and more than 0.01% were detectable. Scatterplots show line at median. Unpaired data were compared with Mann-Whitney-test. P<0.05 was considered significant, only significant p values are documented in the figures.
